# Getting hands on a drug for Covid-19: Inhaled and Intranasal Niclosamide

**DOI:** 10.1016/j.lanepe.2021.100094

**Published:** 2021-04-13

**Authors:** Karl Kunzelmann

**Affiliations:** Physiological Institute, University of Regensburg, University street 31, D-93053 Regensburg

The SARS-CoV-2 (severe acute respiratory syndrome coronavirus 2) pandemic has caused more than 2.6 million deaths as for March 16th 2021 (https://coronavirus.jhu.edu). Viral vector and mRNA vaccines have been quickly developed and are now broadly used around the globe to master this global challenge. However, it is unlikely that vaccination alone will curb the COVID-19 pandemic, therefore asking for additional therapeutic and prophylactic approaches.

Backer and colleagues performed the first randomized, double-blind, placebo-controlled phase 1 trial of inhaled and intranasal application of the antiviral compound niclosamide [1]. Niclosamide is on the WHO list of essential medicines and is already used for decades as an oral anthelminthic. Earlier studies *in vitro* and in mice *in vivo* demonstrated a broad potent antiviral effect of niclosamide against SARS-CoV-2 and other viruses [2]. Backer et al. designed a new formulation for niclosamide, named UNI91104, which allowed for a highly concentrated stock solution optimized for inhalation and nasal application. Fifty-four healthy volunteers were randomly assigned to ascending single doses or alternatively five repetitive doses up to 50.4 mg over 2.5 days. Inclusion criteria included a minimum of 80% predicted lung function. Major exclusion criteria included severe clinically relevant allergies, airway diseases, and other current acute or chronic conditions. As safety assessment possible adverse effects were recorded, using lung function tests like forced expiratory volume in one second (FEV1) and fractional exhaled nitric oxide (FeNO). The frequency of adverse effects and their dependence on dose were used as primary endpoints. No serious adverse events or discontinuation were reported. Most frequent was a mild irritation of upper airways in 59%, signs of increased FeNO in 14.7% and an asymptomatic drop in FEV1 in 11.8%.

The present study has some limitations due to the small sample size, the very short period of treatment, and the exclusion of patients with diagnosed respiratory comorbidities such as asthma or COPD. If niclosamide will be used as prophylactic and therapeutic treatment for Covid-19, long term application as well as effects in patients with pulmonary symptoms need to be evaluated. A rather positive finding was the low systemic niclosamide concentration that was comparable to the plasma level reached by a much higher oral dose of 2 g, used for anthelminthic treatment. Therefore, topical aerosol application of niclosamide might be the way to go, as it may produce high local concentrations in oropharynx, upper and lower airways, were the viral burden is the highest. This might turn out to be superior to oral application or intramuscular injection, currently tested in a number of other ongoing clinical trials (clinical trials.gov).

Repurposing of niclosamide has been also proposed for the treatment of other pulmonary conditions, such as asthma [3] and cystic fibrosis [4]. It has potent bronchodilating effects, and inhibits excessive mucus production [5]. Due to its effects on intracellular Ca^2+^ levels, niclosamide also inhibits the release of proinflammatory cytokines such as IL-8, and possibly also other cytokines, which could be of utmost importance to curb the cytokine storm frequently observed in hospitalized Covid-19 patients. Another fortunate aspect is the antibacterial activity of niclosamide that could be most welcome in fighting potential pulmonary superinfections [6].

Without doubt, the present paper is of large significance to clinicians and health policy makers due to the urgent need for a drug to treat SARS-CoV-2 infections. Although the UK and USA monitoring systems, Yellow card and VAERS do not show an unusual number of adverse reactions or fatal outcome by Sars-Cov-2 vaccines [7,8], there are serious issues related to safety and decreased efficacy of the vaccines in older patients [9], allergic reactions and antibody-dependent enhancement potentially causing cytokine storm [8,10]. Repurposing of what is already available in combination with topical rather than systemic application to minimize unwanted side effects, might be the way to go. Without an effective drug, the death toll and huge socioeconomic costs of this pandemic will continue to increase. A drug is urgently needed, as it becomes more and more obvious that vaccination alone might not sufficient to curb the pandemic caused by this rapidly mutating virus. A cheap and well explored drug like niclosamide could be the way to go.

## Contributors

Karl Kunzelmann wrote commentary

## Funding

DFG-KU756/14–1

## Declaration of interests

KK has nothing to declare and no competing interests. Patent application by the University of Regensburg, 93053 Regensburg, Germany. PCT/EP2019/077433: COMPOUND FOR USE IN THE TREATMENT OF A DISEASE CHARACTERIZED BY DYSREGULATED MUCUS PRODUCTION AND/OR SECRETION.
